# Stimulant abuse as a coping strategy—Forensic and criminal consequences of stimulant abuse for neuroenhancement

**DOI:** 10.3389/fpubh.2022.1028654

**Published:** 2022-10-28

**Authors:** Philipp Dominik, Martin P. Waßmer, Michael Soyka, Andreas G. Franke

**Affiliations:** ^1^Institute for Criminal Law and Criminal Procedure Law, University of Cologne (UoC), Cologne, Germany; ^2^Department of Psychiatry and Psychotherapy, Ludwig Maximilian University (LMU), Munich, Germany; ^3^University of Applied Labour Sciences, Mannheim, Germany

**Keywords:** cognitive enhancement, criminal law, punishment, stimulants, legal situation, fine, neuroenhancement

## Abstract

Pharmacological neuroenhancement (PN) describes the use of divergent psychoactive substances to enhance mental performance (cognition) without medical need. This kind of substance abuse takes place predominantly in stressful situations. Users implicitly—or even explicitly—describe this kind of drug abuse to be a coping strategy. Regarding the decision making process whether to use PN drugs or not, users indicate that legal aspects to be decisive. However, the legal situation has been neglected so far. To elucidate the German legal situation, PN substances have to be divided into over-the-counter drugs, prescription drugs and illegal drugs. Amphetamines have the highest cognition-enhancing potential, followed by modafinil and caffeine-containing substances. It is pointed out that the use of both freely available and prescription PN substances and narcotics without medical indication have so far been largely exempt from punishment under German law. However, individuals (physicians, bus and truck drivers, etc.) taking PN substances may expose others at risk due to wrong decisions (driving or treatment), errors based on side effects of the used substances. Therefore, the protection of life and health of others could legitimize criminal regulation.

## Introduction

The age-old dream to increase human performance seems to become true for mental performance due to recent pharmacological developments. Mental performance enhancement is described with various terms such as “Pharmacological Neuroenhancement” (PN), “Brain Doping,” or “Academic Performance Enhancement” (1–3). The corresponding substances are often referred to as “smart drugs” (1, 4). Except from technically elaborated experimental approaches such as (magnetic) brain stimulation *via* huge laboratory “machines,” the pragmatic and easier way to increase mental performance is delivered *via* the use of substances (5–7). Some of these substances are well-known to be drugs of abuse and addiction.

Klaus Lieb and Andreas G. Franke and the many other scientists belonging to the research field of PN define the latter as the use of specific substances by healthy individuals to increase mental performance without any medical need and therefore without any medical indication (5–8). Specific aims of this use are to increase cognitive domains such as vigilance, attention, concentration and memory. However, even the attempt to increase motivation and mood, or attempts to appear more intelligent to others or even to “be smart” falls under the definition of PN. The latter is sometimes also referred to as mood enhancement (3).

The motivation for the use of PN substances usually originates in the desire for a more intensive use of one's leisure time, which is aimed to be achieved by attempting to reduce or condense working hours and achieving (professional) goals in a shorter time (9) being underlined by divergent studies (6, 10–13). Therefore, PN is often considered to be a coping strategy that has been underlined in national (Germany) and international studies (13–21)—a coping strategy by the use of drugs that can lead to abuse and/or addiction.

Multiple studies underline the character of PN to be a coping strategy. A representative survey in Germany examined the professional situation of workers (14). It showed that participants felt psychological stress from the “circumstances” of their work (e.g., workload, multitasking, pressure to perform, etc.). Furthermore, data was collected on recovery strategies showing an increasing demand both in a professional and private environment (15). Eight percent of the surveyed 2,000 participants reported using prescription drugs to “unwind” and 12% to cope with the needs mentioned above.

Already in 2009, one of the largest German health insurance companies introduced this topic with a focus report on “Doping at work” (22, 23): According to the report of 2009, 5% and in 2015 even 6.7% of the surveyed employed persons between 20 and 50 years of age admitted to use prescription drugs to enhance cognitive performance or mental wellbeing without indication. In addition, many of the surveyed participants stated that PN was justifiable for occupational needs. The authors of the study estimated the number of unreported cases at 12.1%.

Studies using survey techniques to increase anonymity (internet-based, anonymization techniques) show prevalence rates of up to 20% use of prescription and illicit drugs among students, surgeons, “white collar workers” and readers of Nature magazine (6, 12, 24, 25). In addition, several studies showed that PN substance use is a coping strategy for perceived stress (12, 26, 27).

Interestingly, an older study demonstrated clearly that legal aspects such as punishment are crucial for the decision whether or not to use prescription and/or illegal PN medicines (28). However, the legal situation in Germany has not been analyzed systematically so far.

Therefore, aim of the paper was to (1) review potential and frequently used drugs for PN, (2) demonstrate their clinical and especially pro-cognitive effects and side effects and (3) integrate these drugs in legal categories based on the German legal system. Thus, an integration of PN substance use in legal categories was the predominant aim of this review. To achieve this aim systematically, the manuscript is designed according to the PRISMA guidelines.

## Materials and methods

To examine the three above mentioned goals, an intensive study of the literature was performed.

Two of the authors (raters) performed a literature search with the search terms “neuroenhancement,” “cognitive enhancement,” “academic performance enhancement” and “smart drugs” in the natural science data base “pubmed.”

For filtering and to reduce the number of hits, quotation marks were used. Then, only hits were used having an abstract and a full text. For further specification, only studies were included that were published later then 2007. To answer aspect (a) (types of substances) specifically and to address only the above mentioned definition by Lieb and Franke as well as many other scientists investigating PN, all studies about drugs affecting mood and/or moral were excluded. Furthermore, as well as regarding the second aim (b) (pro-cognitive effectiveness), only studies were included that demonstrate pro-cognitive effects and side effects; to explain effects on the molecular level, references were chosen voluntary. In a second step all abstracts were screened by two raters and publications which did not meet the subject (a, b, or c) sufficiently were excluded.

Because a plethora of studies from the field of natural sciences was found and to explain the subject to the reader in a reasonable and comfortable way, only a convenience sample characterizing and demonstrating PN drugs robustly, was cited in this article.

For studying the third aim (c) to integrate and characterize use of PN drugs with respect to legal categories, the German legal databases “juris” and “beck-online” were used and only studies were included which were published after 2008. Therefore, two authors performed a literature search using the search terms “neuroenhancement,” “Selbstdoping,” and “mentale Selbstbestimmung.” Because of different legal systems all over the world, the German situation is not comparable to others. To characterize the latter, only German speaking databases have been searched for legal sources.

Subsequently to the literature search, search results of the two authors were compared and discussed.

## Results

**Figure 1:**
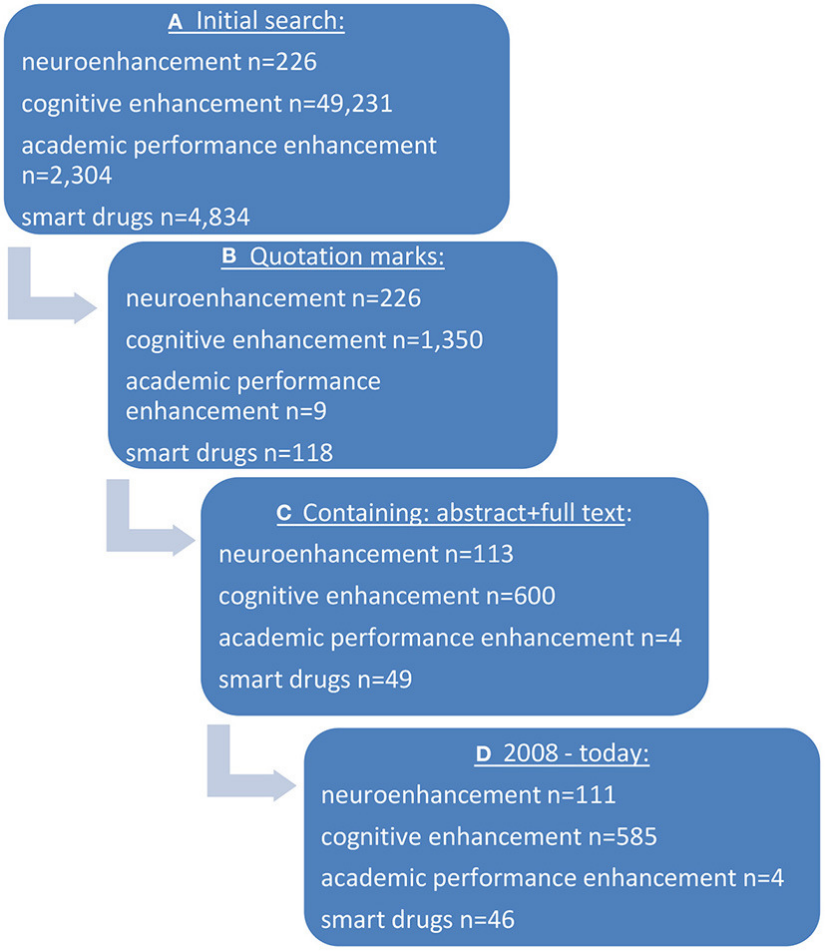
Search strategy in “pubmed” for **(A)** defining substances and **(B)** show clinical effects. **(A)** Initial search terms were used, **(B)** quotations marks were added, **(C)** only publications were used with abstracts and full text, **(D)** only publications published later than 2007 were considered.

**Figure 2:**
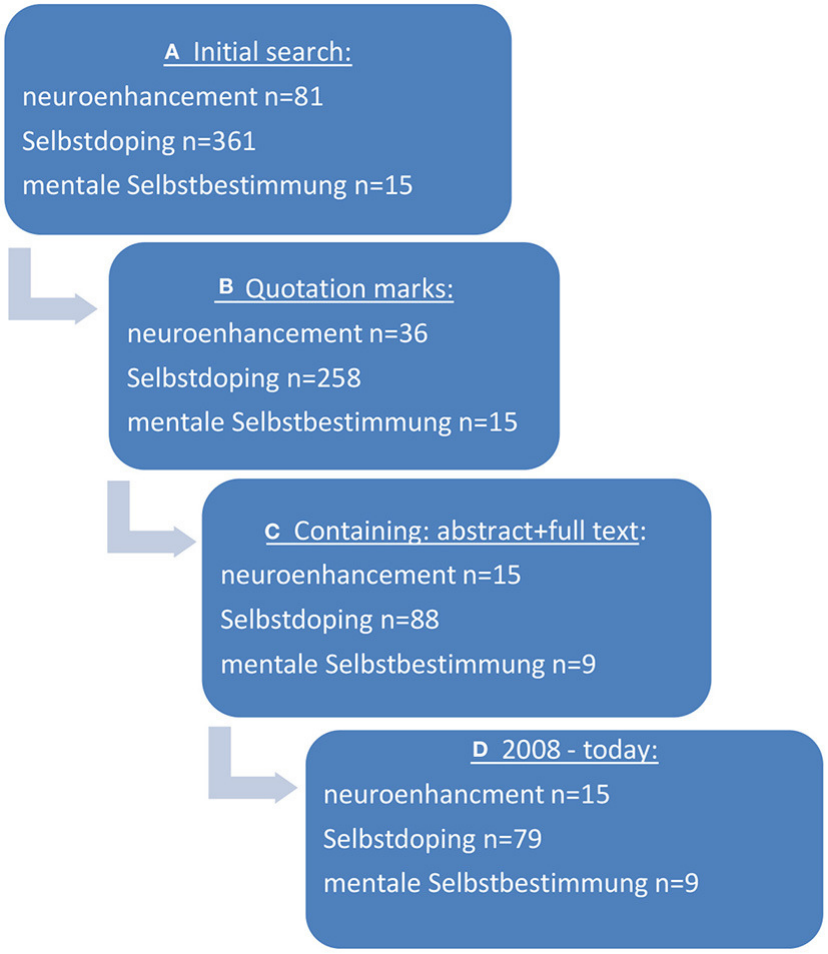
Search strategy in “juris” and “beck-online” for the search terms “neuroenhancement,” “Selbstdoping,” and “mentale Selbstbestimmung” to find data about legal aspects of PN. **(A)** Initial search terms were used, **(B)** quotations marks were added, **(C)** only publications were used with abstracts and full text, **(D)** only publications published later than 2008 were considered.

When performing the initial search in “pubmed” in sum, *n* = 56,595 hits/papers were identified using the specific search terms (neuroenhancement, cognitive enhancement, academic performance enhancement, smart drugs). Using filters (quotation marks, only papers containing an abstract as well as the full text, papers between 2008 and today) hits were reduced to *n* = 746 publications. After having screened the respective abstracts by two raters for the question of being relevant for the subject, *n* = 322 publications remained. For further details see Figures 1, 2.

### Categories of PN substances

Substances used for PN purposes are a heterogenous group of different substances. Therefore, today's PN drugs can be categorized in different ways (13, 29–31). The following categorizations are based on substance characteristics (13, 31–33) and are used by several scientists with an emphasis on a European and especially German speaking field (3, 8, 11, 13, 25): (1) prescription drugs, (2) illegal drugs, and (3) freely available over-the-counter (OTC) substances or (1) stimulants and (2) non-stimulants (see Table 1) (8). These and other categorisations have been developed years ago. However, they are referred to for PN substance research.

**Table 1:** Possible classifications of CE drugs.

**(A) GENERAL CLASSIFICATION**	
Over-the-counter drugs (OTC drugs)	• Methylxanthines: caffeine and caffeinated drinks (coffee, energy drinks), caffeine tablets. • Herbal medicines: ginkgo biloba, ginseng, etc. • Lifestyle and vitamin supplements: Vitasprint^®^, Dextro Energy^®^, etc. • Homeopathic substances/preparations
Prescription substances	• Prescription stimulants underlying the German Narcotics Act being marketable: methylphenidate (e.g., Ritalin^®^), amphetamines (e.g., Attentin^®^). • Prescription non-stimulants that are not subject to the German Narcotics Act being marketable: modafinil, anti-dementia drugs, antidepressants, beta-blockers, benzodiazepines, etc.
Illegal substances	• Illegal stimulants underlying the German Narcotics Act being not marketable: amphetamines (e.g., “speed,” “ecstasy“), etc.
**(B) CLASSIFICATION DUE TO ADDICTION MEDICINE**	
Stimulants	• Methylxanthines: coffee, caffeine containing beverages and foods, caffeinated/energy drinks, and caffeine tablets • Prescription stimulants underlying the German Narcotics Act being marketable: methylphenidate (e.g., Ritalin^®^), amphetamines (e.g., Attentin^®^, Adderall^®^). • Illegal stimulants being governed by the German Narcotics Act being not marketable: amphetamines (e.g., Speed, Ecstasy)
Non-stimulants	• Prescription non-stimulants, not underlying the German Narcotics Act being marketable: Modafinil, antidementia drugs, beta-blockers, benzodiacpines, etc. • Phytopharmaceuticals: Ginkgo biloba, Ginseng, etc. • Lifestyle and vitamin “drugs,” e.g., Vitasprint^®^, Dextro Energy^®^, etc. • Homeopathic substances/preparations
**(C) LEGAL CLASSIFICATION**	
Narcotics	• Prescription stimulants underlying the German Narcotics Act being marketable: methylphenidate (e.g., Ritalin^®^), amphetamines (e.g., Attentin^®^). • Illegal stimulants underlying the German Narcotics Act being not marketable: amphetamines (e.g., “speed,” “ecstasy“), etc.
Other substances	• Prescription non-stimulants not underlying the German Narcotics Act being marketable: modafinil, antidementives, beta-blockers, benzodiacepines, etc. • Methylxanthines: caffeine, caffeinated drinks (coffee, energy drinks), caffeine tablets. • Phytopharmaceuticals: Ginkgo biloba, ginseng, etc. • Lifestyle and vitamin supplements, e.g., Vitasprint^®^, Dextro Energy^®^, etc. • Homeopathic substances/preparations

Considering the above mentioned German categorisations by Lieb and Franke the subdivision into stimulants and non-stimulants, methylxanthines such as caffeine (coffee, caffeinated drinks/energy drinks, and caffeine tablets) make up the greatest percentage of the available stimulants (8). However, stimulants such as amphetamines and cocaine also belong to this group. Whilst caffeine is freely available and contained in various foods and beverages, the latter (amphetamines) are subject to the German Narcotics Act. This group of stimulants has to be divided into the further subgroups illegal (e.g., speed and ecstasy) and prescription stimulants (e.g., Attentin^®^ and Adderall^®^), as well as the derivative methylphenidate (MPH, e.g., Ritalin^®^). In comparison to these cocaine plays an insignificant (rarely used) role. The group of non-stimulants consists of prescription drugs such as anti-dementia drugs and OTC substances; these are mainly phytopharmaceuticals such as Ginkgo biloba (for neurobiological details see (33).

From a national (German) legal perspective, a new categorization is mandatory which is based on the German Narcotics Act (Betäubungsmittelgesetz—BtMG) is to be made according to narcotics and other substances (see Table 1C). This is appropriate, as the abuse of a substance is only relevant under criminal law if the substance is covered by the law. Otherwise, due to the principle of “nulla poena sine lege,” which is constitutionally anchored in Article 103 (2) of the German Basic Law (Grundgesetz—GG) and in § 1 of the German Criminal Code (Strafgesetzbuch—StGB), punishment is excluded.

### Clinical effects of PN substances

Literature search revealed that measurement for effect sizes was not possible. The present studies used a plethora of different methods to measure (pro-) cognitive effects.

#### Caffeine

Caffeine is the most common substance used for PN. Although coffee is considered a “normal” part of everyday life (e.g., “coffee break”) and a stimulant, it could be argued that caffeine is not a “true” PN substance. However, caffeine belongs to the group of methylxanthines that have pro-cognitive effects based on randomized control trials (RCTs). Furthermore, in numerous survey studies, the consumption of coffee is rated as PN consumption behavior e.g., (34–38). In addition to coffee, caffeine-containing foods and beverages, including so-called energy drinks (e.g., Red Bull^®^), are sources of caffeine that are marketed specifically for “modern” consumption. Energy drinks promise the consumer “energizing” effects (39).

Furthermore, Coffeinum^®^ is the only caffeine-containing drug approved for short-term fatigue. Although a prescription is not required, Coffeinum^®^ is only sold in pharmacies and not in supermarkets or drugstores.

The clinical effects of caffeine include tachycardia, hypertension, and bronchial dilation (40). At least in part, these effects may explain the pro-vigilant effect of caffeine. RCTs with different doses of caffeine (50–600 mg) in non-sleep-deprived subjects show that vigilance, attention, concentration and psychomotor activity are increased by the consumption of energy drinks. In sleep-deprived subjects treated with 150–600 mg of caffeine, the effects are even more pronounced. However, the available RCTs show only few and inconsistent effects of caffeine on higher cognitive domains (e.g., memory) (8, 41, 42).

Interestingly, energy drinks (250 ml, 80 mg caffeine, 1.000 mg taurine) seem to have stronger clinical effects than the same dose of “pure” caffeine without other ingredients (8). The reason for this is still unclear but may be due at least in part to a specific marketing strategy (39). A study among surgeons concludes that drinks containing caffeine and taurine bring simulated laparoscopic performance back to the level of the more or less well-rested state. However, a reduction in surgeon error rate was not observed (43).

Compared to other PN substances, the cognition-enhancing effects of caffeine are roughly comparable to the effects of MPH and modafinil (44–46). However, this is dose-dependent. A study among healthy chess (tournament) players shows the following order of effectiveness: methylphenidate > modafinil > caffeine (47).

#### Amphetamines and methylphenidate

Methylphenidate (MPH, e.g., Ritalin^®^) and amphetamine products such as Attentin^®^ or Adderall^®^ being a mixture of amphetamine and dextroamphetamine are mainly approved for the treatment of attention-deficit/hyperactivity disorder (ADHD). In addition, AMPHs belong to the group of illegal drugs (e.g., “speed,” ecstasy). Patients need a special prescription under the German Narcotics Act (BtMG) to take drugs containing MPH and AMPH.

RCTs show that AMPHs and its derivative MPH increase vigilance and attention in healthy volunteers, leading to a shortened reaction time. Comparable to those of caffeine, the effects of these drugs are enhanced in sleep-deprived healthy volunteers (3, 8, 48). Similar to caffeine, these enhanced pro-cognitive effects in sleep-deprived participants may lead to the assumption that the pro-cognitive effect on higher cognitive domains such as memory is an indirect effect mediated by an increase in vigilance, attention and concentration (3, 8). However, there are few data that attempt to demonstrate that the pro-cognitive effects are independent of the waking state (sleep-deprived vs. non-sleep-deprived) (49).

A systematic review of MPH in relation to PN in healthy subjects found that the pro-cognitive effect of MPH is difficult to assess due to the heterogeneity of studies and neurocognitive tests used. However, the review found that MPH improved cognitive performance on novel tasks and attention-based tasks and reduced planning latency on more complex tasks (50).

Compared to MPH, AMPH have pronounced effects on vigilance, attention and reaction time possibly due to the mode of action (8). Higher doses of stimulants lead to euphoric effects or at least a better mood (48). In this regard, abuse and dependence on AMPH have long been controversially discussed (51–53).

#### Modafinil

Modafinil (e.g., Vigil^®^) is licensed for the treatment of narcolepsy with excessive daytime sleepiness. Depending on the country and its specific regulations, modafinil does (not) require a specific prescription. The clinical effects of modafinil are similar to those of AMPH and MPH leading to increased vigilance, attention and shortened reaction time (8, 48). However, the above-mentioned study among healthy (tournament) chess players shows that MPH has a higher efficacy than modafinil (47). However, interestingly one most recent review denies the pro-cognitive potential of Modafinil (54).

After sleep deprivation, the above-mentioned pro-cognitive effects are pronounced compared to the effects of modafinil in rested subjects (8, 48). A meta-analysis shows a large research gap regarding PN by modafinil. However, using an extensive neurocognitive test battery, the authors show enhancing effects on a few cognitive domains, such as working memory, attentional interference, spatial planning and executive functions (55). An older systematic review shows that modafinil improves attention even in well-rested individuals. However, maintenance of vigilance, memory and executive functions were improved to a significantly greater extent in sleep-deprived individuals. Repeated administration of modafinil did not prevent deterioration in cognitive performance over a prolonged period of sleep-deprivation (49). An older study examined therapeutic doses of modafinil during simulated night shifts and showed attenuation of the cognitive decline associated with night shifts (56). Another important study, using the CANTAB comprehensive neurocognitive test battery and an artificial surgical (laparoscopic) simulation setting, showed that modafinil led to improvements in working memory and planning behavior, a reduction in impulsivity and increased flexibility (57).

In terms of comparison with other stimulants, RCTs show that the pro-cognitive effects of modafinil is equivalent to that of stimulants and caffeine when 400 mg of modafinil is used compared with 20 mg of d-amphetamine and 600 mg of caffeine (44, 45, 58). It should be stressed that 600 mg of caffeine are a very high dose, equivalent to six cups of coffee or three tablets of Coffeinum^®^. The latter is approved for a maximum of 400 mg per day. Beyond that, an aviation setting seem to underline the results from the chess study mentioned above (47, 48), however, another comparison using an extensive test battery denies the efficacy of modafinil (46).

Beyond that, there seem to be a certain abuse potential or even risk of addiction (54).

#### Antidementia drugs

The group of antidementia drugs consists of two different mechanisms of action and thus two different types of drugs. Both types of drugs are approved for the treatment of Alzheimer's disease only.

The available studies on antidementives hardly allow for comparisons between the aforementioned antidementives, as the study designs pursue other goals besides PN (45). Moreover, the sparse results of RCTs on PN are inconsistent (3, 8). There is even some evidence of worsening of reaction time and memory by antidementives. RCTs with healthy subjects show that antidementia drugs do not have consistent effects on cognition, neither in terms of vigilance and attention, nor in reaction time or memory (3, 8).

Apart from these study results, antidementia drugs have side effects that are very common (59).

#### Ginkgo biloba

Ginkgo biloba with antioxidant characteristics of leaves of the Ginkgo tree is used to prevent dementia and when cognitive deficits occur. It is also used to increase cognitive abilities in a population of people over 60 years of age with a high level of education. In this group and for the above-mentioned “indications,” the prevalence is 15% (60).

According to the SmPC (“summary of product characteristics,” also known as medication package insert), in which all aspects of a medicinal product must be described in accordance with regulatory concerns, Ginkgo biloba is approved for peripheral arterial occlusive disease, dizziness of vascular and involutive origin, as well as tinnitus and the so-called “dementia syndrome.” The latter is not synonymous with the term “dementia” and does not correspond to the medical term and definition of “dementia.”

Pro-cognitive effects could not be proven in laboratory studies (61) in accordance with missing clinical effects based large Cochrane meta-analyses; however, there are (almost) no side effects (62, 63). Another meta-analysis came to the conclusion that Ginkgo biloba must be dosed with at least 240 mg per day. This dosage seems to lead to clinical stabilization in demented patients as well as in people with mild cognitive impairment. In addition, ginkgo biloba seem to have the potential to reduce progression of cognitive impairment (64). A most recent “narrative review” does not come to a clear conclusion describing confusing results of divergent studies with different Ginkgo extracts (65).

Ginkgo biloba seems to be an interesting candidate for PN, as it has (almost) no side effects, but little or questionable pro-cognitive effects.

### Forensic psychiatric aspects

Drug abuse and addiction play a very large role with respect to aggressive and antisocial behavior, delinquency and acquisitive crime. Many drug users become delinquents. Research has largely focused on opiate addiction. It has been shown that about 20% of prisoners in Germany are addicted to opiates (66). A survey in Berlin prisons showed a prevalence rate of 16% for opiate addiction (67). Similar figures of 18–23% were reported for the USA (68).

The use of psychostimulants, which can lead to disinhibition and aggression, could be forensically relevant. The induction of psychotic and manic states is even reported as a potential side effect in drug product information for psychostimulants.

### Criminal legal evaluation

The fact that active substances, which are used for therapeutic purposes only (69), are also used by healthy people for cognitive performance enhancement leads to the question of the assessment of this scenario under criminal law. So far, PN has not been subject to by special regulation in Germany. Therefore, an overview will be given below as to whether the existing norms adequately cover PN use.

As a rule, criminal law can only threaten consequences if a norm of the German core criminal law [German Criminal Code (StGB)] or the supplementary criminal law [criminal norms in other laws such as the German Narcotics Act (BtMG)] makes an act punishable. A fine or prison sentence can only be imposed if an offender commits an act that is punishable at the time of the offense.

The German core criminal law is not applicable to the consumption of any of the substances mentioned above. Especially substances that are freely marketable within the legally prescribed potencies, such as caffeine, taurine, Ginkgo biloba, ginseng, etc., are not covered as the clinically “harmless” effect of these “everyday” preparations is socially accepted. The same applies, for example, to anti-dementia drugs—although these are only available on prescription. Moreover, they are not considered narcotics.

Substances such as AMPH, MPH or cocaine are subject to the German supplementary criminal law due to their significantly higher risk potential (MPH and cocaine in Schedule III and methamphetamine in Schedule II of the German Narcotics Act). However, the consumption as such is not made a punishable offense thereunder either.

#### More detailed explanations on self-enhancement

From a legal perspective, medicine is facing a profound structural change. Alongside the traditional, altruistic view of medicine's raison d'être, such as healing, palliation or accompaniment, a modern (wish-fulfilling) medicine has developed. Physicians embellish, improve and optimize people (70). The medical ethics enshrined in the Hippocratic Oath prescribe helping the sick (71). The improvement of healthy people leads this maxim ad absurdum.

#### Bodily injury according to § 223 of the German Criminal Code (StGB)

Although some of the drugs under investigation cause quite considerable side effects, the use of PN is not punishable as a form of bodily harm. The basic offense [§ 223 (1) of the German Criminal Code (StGB)] requires (1) physical abuse or (2) damage to the health of another person (72). If someone harms themselves by consuming PN, no harm is done to others. According to the established case law of the Federal Supreme Court, self-inflicted self-harm is not punishable (73). Punishment would not be compatible with the German Basic Law (GG). The impunity of self-harm is a consequence of the right to self-determination, which is guaranteed in Article 2 (1) in conjunction with Article 1 (1) of the German Basic Law (74).

#### Negligence offenses

A physician who takes PN to improve his performance is not normally liable to prosecution for a negligence offense (especially negligent bodily injury or homicide). This is because the user assumes that he will improve his own performance by taking it. If he has not had any negative experiences in the past (coordination problems, overestimating himself, etc.), he does not violate his duty of care to treat a patient only if one is physically able to do so. On the contrary, he wants to improve the treatment of his patients.

If an injury nevertheless occurs that can be causally attributed to the substance use, it must usually be assumed that the physician, according to his personal abilities and the degree of individual skill at the time of use, could not have recognized that he was endangering the patient (75).

#### Fraud according to § 263 of the German Criminal Code (StGB)

The acquisition of PN is also regularly not punishable as fraud. However, many case scenarios are imaginable, therefore no generally applicable result can be formulated.

In most cases, there is no “fact” which would be relevant in the context of fraud (76) about which the user deceives his counterpart (77) and thereby arouses or maintains a misconception (78). Typically, it is assumed that the counterpart has no knowledge that the performance has improved pharmacologically. Mere ignorance of the facts (79) (“ignorantia facti”) does not regularly constitute a misconception. The absence of a conception does not constitute an error; such an error only arises from a misconception (80).

Irrespective of this, there is no causal damage. The employment relationship can be cited as an example. The employer undertakes to pay the agreed remuneration pursuant to § 611a (2) of the German Civil Code (Bürgerliches Gesetzbuch—BGB). If he hires an employee, damage could already occur at the time of the conclusion of the employment contract (81), “if the value of the claim to the work performance falls short of the remuneration agreed for it” (77). Ultimately, however, it must be countered that such damage is of a purely fictitious nature. The criminal law requirement of certainty derived from Article 103 (2) of the German Basic Law (GG) (82) requires that any damage be quantifiable. Moreover, even an average employee could temporarily underperform in comparison to his usual capacity shown in the application procedure. Especially in the field of human performance, a generally valid quantification is hardly possible.

#### German Anti-Doping Act

Since the introduction of the German Anti-Doping Act (Anti-Doping-Gesetz—AntiDopG) on 10 December 2015, the area of sport has been regulated. § 3 para. 1, § 4 para. 1 no. 4 of the German Anti-Doping Act punish the athlete who seeks to gain an advantage in a competition of organized sport by taking performance-enhancing substances (83). Performance-enhancing substances are those listed in Annex I of the International Convention against Doping in Sport of 19 October 2005. For the substances considered here, this means that amphetamine, methamphetamine, cocaine, MPH, modafinil and active substances with a similar chemical structure or similar biological effect(s) are covered.

#### Interim result

It should be noted that self-enhancement is only punishable under the German Anti-Doping Act.

#### Other offenses

##### German Narcotics Act (BtMG)

According to § 29 (1) of the German Narcotics Act (BtMG), anyone who illicitly cultivates, manufactures, traffics in, imports, sells, dispenses or otherwise puts into circulation, acquires or otherwise obtains narcotic drugs (No. 1), possesses (No. 3), prescribes, administers or hands them over for immediate consumption (Nos. 6, 6a), provides false or incomplete information in order to obtain a prescription for a narcotic drug for themself or for another person (No. 9) or provides or gives another person an opportunity for the unauthorized acquisition or unauthorized supply of narcotic drugs, communicates such opportunity publicly or selfishly or induces another person to the unauthorized consumption of narcotic drugs (No. 10) is punishable.

##### Unauthorized prescription by a physician

§ 29 (1) sentence 1, no. 6 of the German Narcotics Act (BtMG) punishes the prescription, administration or handover of narcotics for immediate use, when done so contrary to § 13 (1) (84). § 13 (1) of the German Narcotics Act stipulates that the narcotics listed in Schedule III may only be prescribed if their use on or in the human body is justified. The German Narcotics Act conclusively defines when an application is unjustified. This is the case if the purpose intended by the use can also be achieved in another way. Case law has concretised this to the effect that a prescription is only permitted in cases where the use on or in the human body is clearly medically justified (85). Consequently, the physician is obliged to first make a diagnosis that leads to a corresponding indication (84). An application is therefore only justified “if the remedy is suitable as a remedy for the patient's suffering according to the general or by far the predominantly recognized rules of medical science” (85). The prescription may only be made if the physician is convinced after a thorough examination that the application is permissible and advisable according to the recognized rules of medical science (86). This is the case if it promises a healing success (85).

##### Unauthorized trafficking under the German Medicines Act (AMG)

According to § 95 (1) no. 4 alt. 1 of the German Medicines Act (Arzneimittelgesetz—AMG), it is a punishable offense to trade in prescription drugs without authorization. Methylphenidate, cocaine, amphetamine or similar pharmacological neuroenhancers already fall under the German Narcotics Act, whereas the active ingredient modafinil is “only” available on prescription and is not a classic narcotic. Nevertheless, illicit trafficking is punishable. In addition to the professional or commercial supply of prescription medicines to consumers (*via* the distribution channel of pharmacies), the prohibition of unlawful trafficking covers any supply by others against payment (87). The term “trafficking” is primarily used in the German Narcotics Act which the German Medicines Act is based on (88). Thus, every acquisition process is covered (89, 90).

Clinical experience shows that physicians and other health care professionals sometimes use psychostimulants (12). This is problematic because addicted physicians hardly fulfill the requirements for maintaining their license to practice (91).

## Discussion

The “everyday” substances and OTC drugs taken for PN are mostly “harmless” and do not significantly improve brain function (59). From the legislator's point of view, prescription substances that are not subject to criminal law do not have the required degree of danger. Therefore, these substances have to be excluded from further consideration.

Further substances, however, have to be considered even if their clinical effects by influencing mood states and indirectly cognitive functions, are low. The effects of narcotics go beyond this; they increase attention and promote sustained concentration. However, even this is not sufficient to derive punishability from it. It remains that the consumption of substances—outside the scope of the German Anti-Doping Act (AntiDopG)—is exempt from punishment.

This result may displease the interested reader. On the one hand, almost all acts surrounding and leading up to consumption are punishable, merely the consumption itself is not. This appears to be a legal subtlety. However, this is not the case: acts outside of consumption regularly endanger third parties. For example, the trafficking of narcotics endangers the health of third parties. As the state is also tasked with the protection of “public health,” according to Article 2 (2) of the German Basic Law (GG), the application of criminal law is legitimate. If the consumer only harms himself, there is no legitimizing reference to third parties.

This means to the German population that the “only” offense is the ownership of narcotics without having a respective prescription. Of course, using PN drugs may lead to the fact of changes regarding mental states and/or mood. Furthermore, it may lead to aspects of self-harm; however, this is not prohibited by any German law and therefore cannot be punished. Beyond that, it has to be stated that using PN drugs without affecting others, the use seem to be legally “unproblematic.”

This use becomes problematic, when others could be or even are threatened. Therefore, you have to ask what would the criminal law assessment look like if performance-enhancing substances were taken in situations with a third party reference? If a physician took substances after a tiring shift in order to be able to perform an operation with the required attention? Superficially, he is harming his own health. However, performance-enhancing narcotics such as prescription and illegal stimulants are known to cause deficits in judgment, perceptual changes, mental health changes etc. that has to be considered as side effects in the medical understanding given in the respective information (Arzneimittelfachinformation) (92). If a physician operates in this state, a danger to patients can no longer be objectively ruled out. However, if the physician overestimates his abilities, it can usually be assumed that he firmly trusts that he can treat the patient better because of the consumption. With regard to the Hippocratic Oath, it is to be assumed that he acts in the patient's best interest and does not intend to harm the patient's health beyond the necessary invasive measures. According to the current legal situation, he should remain unpunished. However, it should be noted that the circumstances of the “individual case” are always decisive (93). What is meant by individual case? The individual case depends i.a., on the drug (drug characteristics), the amount (dose) and the state of the use [tired, sleep-deprived, etc.; e.g., (44, 47, 58)]. These are the most important aspects determining the “individual case” leading to a more or less significant cognitive and mood change. The consequence as a more or less significant change of behavior and a more or less significant risk of damage for a patient or another person interacting with someone influenced by the use of a PN drug.

Beyond that, it could be argued about the possibility of impairment of the user. However, PN drugs are not known to decrease the users' level of functioning. Therefore, this aspect does not have to be considered.

Except from the above mentioned physician, easier and less complicated cases are imaginable e.g., nurses, taxi, bus or truck drivers, pilots or other people (in a professional or non-professional context) demonstrating the possibility to harm others influenced by the use of PN drugs.

From a legal perspective, it must be stated that the consumption of any PN substances is exempt from punishment. Based on the known clinical effects, there seem to be no need for further regulation based on a lack of cases of accidents, deaths, malpractice or even violence in the context of PN. If there are no such cases or the context could not be drawn cannot be evaluated yet.

It is conceivable, however, that research into more effective substances will necessitate a different assessment under criminal law in the future. The jurisprudence is obliged to take medical progress into consideration.

Apart from the clinical effects of PN drug use, there are already weighty reasons in favor of criminal law regulation. Because of the more or less rare side effects (e.g., mania, psychosis, overconfidence, euphoria, etc.) physicians and others (nurses, truck drivers, etc.) can endanger the physical integrity of their patients or others. It is hardly predictable when side effects such as mania, psychosis, overconfidence, euphoria, etc. will set in. The users decisions (speed of driving, using specific surgical instruments, landing a plane, etc.) can be negatively influenced by this. Through PN, users may set others on risk; e.g., the physician exposes his patients to the risk of wrong decisions and treatment errors. He thereby impairs the trust in the physician-patient relationship. The protection of life and limb and the reliability of health care services could legitimize regulation under criminal law.

Bringing together medical and legal aspects of the PN phenomenon systematically for the first time, the manuscript allows a legal approach based on medical and especially pharmacological aspects. However, there are some aspects that are limiting the meaningfulness of this article that have to be considered.

Main problem of this article is the fact that there are more or less different legal systems all over the world. This means that the German legal perspective regarding PN is not transferrable to other countries' legal systems. However, it has to be stated, that there are some fundamental legal principles being equal in many countries' legal systems. Therefore, this review allows a slight approach to an international consideration how to deal with PN drug use. Comparing the legal situation of PN drug use internationally or at least multinationally should be investigated in further studies.

Beyond this major weakness of the paper, the legal consideration is based on a systematic search of the literature in one search engine (pubmed). This search engine is the most well-known and used data base of the field of natural sciences including medicine and pharmacy. However, there are further but smaller and less important data bases. This may have led to the possibility that some studies deriving from the field of psychology are not included in this review. However, the substances that are included in this review are designated in the vast majority of the studies found. The used studies show an enormous unity in designating substances. Only very few studies suggested to add further substances such as beta blockers or benzodiazepines (6). However, the respective studies argued that benzodiazepines and beta blockers are used to get “calm” e.g., in case of stage fright of musicians or to counteract a high arousal during daytime (29, 94). These properties do not meet the definition of PN by Franke and Lieb (3, 8) the review is based on. Therefore, substances such as benzodiazepines and beta blockers are not included in this review.

Beyond that, studies measuring the clinical effects of PN drugs are based on a plethora of assessment instruments. These psychological assessment instruments cannot be compared to each other. Therefore, the review is unable to compare the studies and putative effective sizes directly to each other. However, to gain insight being as objective as possible (primarily), studies have been chosen using a RCT design (randomized, double-blind, placebo-controlled trials) to characterize clinical effects of the PN substances. This RCT study design offers an objective analysis of (clinical) effects avowing any subjective bias. Therefore, the evaluation of clinical effects is based (primarily) on this type of studies.

## Data availability statement

The raw data supporting the conclusions of this article will be made available by the authors, without undue reservation.

## Author contributions

All authors listed have made a substantial, direct, and intellectual contribution to the work and approved it for publication.

## Conflict of interest

The authors declare that the research was conducted in the absence of any commercial or financial relationships that could be construed as a potential conflict of interest.

## Publisher's note

All claims expressed in this article are solely those of the authors and do not necessarily represent those of their affiliated organizations, or those of the publisher, the editors and the reviewers. Any product that may be evaluated in this article, or claim that may be made by its manufacturer, is not guaranteed or endorsed by the publisher.

## References

[1] Nicholson PJ Wilson N. Smart drugs: implications for general practice. Br J Gen Pract. (2017) 67:100–1. 10.3399/bjgp17X689437 28232331 PMC5325623

[2] Partridge BJ Bell SK Lucke JC Yeates S Hall WD. Smart drugs “as common as coffee”: media hype about neuroenhancement. PLoS ONE. (2011) 6:e28416. 10.1371/journal.pone.0028416 22140584 PMC3227668

[3] Franke AG. Hirndoping & Co. Die optimierte Gesellschaft. Heidelberg: Springer-Verlag (2019). 10.1007/978-3-662-58853-6

[4] Dance A. Smart drugs: a dose of intelligence. Nature. (2016) 531:S2–3. 10.1038/531S2a 26934523

[5] Farah MJ. Neuroscience. Unknowns Cogn Enhanc Sci. (2015) 350:379–80. 10.1126/science.aad5893 26494744

[6] Maher B. Poll results: look who's doping. Nature. (2008) 452:674–5. 10.1038/452674a 18401370

[7] Greely H Sahakian B Harris J Kessler RC Gazzaniga M Campbell P . Towards responsible use of cognitive-enhancing drugs by the healthy. Nature. (2008) 456:702–5. 10.1038/456702a 19060880

[8] Franke AG Bagusat C Rust S Engel A Lieb K. Substances used and prevalence rates of pharmacological cognitive enhancement among healthy subjects. Eur Arch Psychiatry Clin Neurosci. (2014) 264(Suppl.1):S83–90. 10.1007/s00406-014-0537-1 25214391

[9] Magnus D. Patientenautonomie im Strafrecht. Tübingen: Mohr Siebeck GmbH & Co. KG (2015). p. 352. 10.1628/978-3-16-153822-3

[10] de Oliveira Cata Preta B Miranda VIA Bertoldi AD. Psychostimulant use for neuroenhancement (smart drugs) among college students in Brazil. Subst Use Misuse. (2020) 55:613–21. 10.1080/10826084.2019.1691597 31790311

[11] Dietz P Iberl B Schuett E van Poppel M Ulrich R Sattler MC. Prevalence estimates for pharmacological neuroenhancement in Austrian University Students: its relation to health-related risk attitude and the framing effect of caffeine tablets. Front Pharmacol. (2018) 9:494. 10.3389/fphar.2018.00494 29946254 PMC6006370

[12] Franke AG Bagusat C Dietz P Hoffmann I Simon P Ulrich R . Use of illicit and prescription drugs for cognitive or mood enhancement among surgeons. BMC Med. (2013) 11:102. 10.1186/1741-7015-11-102 23570256 PMC3635891

[13] Maier LJ Liechti ME Herzig F Schaub MP. To dope or not to dope: neuroenhancement with prescription drugs and drugs of abuse among Swiss University Students. PLoS ONE. (2013) 8:e77967. 10.1371/journal.pone.0077967 24236008 PMC3827185

[14] Polzer Ch Figgen M Seiler K Beerheide E Evers G van Loocke-Scholz A . Belastung – Auswirkung – Gestaltung – Bewältigung. Ergebnisse einer Repräsentativbefragung in NRW. Düsseldorf: Landesinstitut für Arbeitsgestaltung des Landes Nordrhein-Westfalen (2014).

[15] Seiler K Beerheide E Figgen M Goedicke A Alaze F Rack R . Ergebnisse einer Repräsentativbefragung in Nordrhein-Westfalen. Düsseldorf: Landesinstitut für Arbeitsgestaltung des Landes Nordrhein-Westfalen (2013).

[16] Franke AG Roser P Lieb K Vollmann J Schildmann J. Cannabis for cognitive enhancement as a new coping strategy? Results from a survey of students at four universities in Germany. Subst Use Misuse. (2016) 51:1856–62. 10.1080/10826084.2016.1200619 27607062

[17] Jensen C Forlini C Partridge B Hall W. Australian University Students' coping strategies and use of pharmaceutical stimulants as cognitive enhancers. Front Psychol. (2016) 7:277. 10.3389/fpsyg.2016.00277 26973573 PMC4771940

[18] Lucke J Jensen C Dunn M Chan G Forlini C Kaye S . Non-medical prescription stimulant use to improve academic performance among Australian University Students: prevalence and correlates of use. BMC Public Health. (2018) 18:1270. 10.1186/s12889-018-6212-0 30453936 PMC6245847

[19] Sattler S Wiegel C. Cognitive test anxiety and cognitive enhancement: the influence of students' worries on their use of performance-enhancing drugs. Subst Use Misuse. (2013) 48:220–32. 10.3109/10826084.2012.751426 23302063

[20] Sattler S. Nonmedical use of prescription drugs for cognitive enhancement as response to chronic stress especially when social support is lacking. Stress Health. (2019) 35:127–37. 10.1002/smi.2846 30378254

[21] Wolff W Brand R. Subjective stressors in school and their relation to neuroenhancement: a behavioral perspective on students' everyday life “doping”. Subst Abuse Treat Prev Policy. (2013) 8:23. 10.1186/1747-597X-8-23 23777577 PMC3698138

[22] Deutsche Angestellten Krankenkasse. Gesundheitsreport 2009. Analyse der Arbeitsunfähigkeitsdaten. Schwerpunktthema Doping am Arbeitsplatz. Berlin: IGES Institut GmbH (2009).

[23] Deutsche Angestelltenkrankenkasse. Gesundheitsreport 2015. Berlin: IGES Institut GmbH (2015).

[24] Dietz P Soyka M Franke AG. Pharmacological neuroenhancement in the field of economics-poll results from an online survey. Front Psychol. (2016) 7:520. 10.3389/fpsyg.2016.00520 27148128 PMC4835716

[25] Dietz P Striegel H Franke AG Lieb K Simon P Ulrich R. Randomized response estimates for the 12-month prevalence of cognitive-enhancing drug use in university students. Pharmacotherapy. (2013) 33:44–50. 10.1002/phar.1166 23307544

[26] Bagusat C Kunzler A Schlecht J Franke AG Chmitorz A Lieb K. Pharmacological neuroenhancement and the ability to recover from stress - a representative cross-sectional survey among the German Population. Subst Abuse Treat Prev Policy. (2018) 13:37. 10.1186/s13011-018-0174-1 30348181 PMC6198480

[27] Maier LJ Haug S Schaub MP. The importance of stress, self-efficacy, and self-medication for pharmacological neuroenhancement among employees and students. Drug Alcohol Depend. (2015) 156:221–7. 10.1016/j.drugalcdep.2015.09.012 26455555

[28] Franke AG Lieb K Hildt E. What users think about the differences between caffeine and illicit/prescription stimulants for cognitive enhancement. PLoS ONE. (2012) 7:e40047. 10.1371/journal.pone.0040047 22768218 PMC3386931

[29] Daubner J Arshaad MI Henseler C Hescheler J Ehninger D Broich K . Pharmacological neuroenhancement: current aspects of categorization, epidemiology, pharmacology, drug development, ethics, and future perspectives. Neural Plast. (2021) 2021:8823383. 10.1155/2021/8823383 33519929 PMC7817276

[30] Lynch G Palmer LC Gall CM. The likelihood of cognitive enhancement. Pharmacol Biochem Behav. (2011) 99:116–29. 10.1016/j.pbb.2010.12.024 21215768 PMC3114293

[31] Carlier J Giorgetti R Vari MR Pirani F Ricci G Busardo FP. Use of cognitive enhancers: methylphenidate and analogs. Eur Rev Med Pharmacol Sci. (2019) 23:3–15. 10.26355/eurrev_201901_16741 30657540

[32] Mehlman MJ. Cognition-enhancing drugs. Milbank Q. (2004) 82:483–506. 10.1111/j.0887-378X.2004.00319.x 15330974 PMC2690227

[33] de Jongh R Bolt I Schermer M Olivier B. Botox for the brain: enhancement of cognition, mood and pro-social behavior and blunting of unwanted memories. Neurosci Biobehav Rev. (2008) 32:760–76. 10.1016/j.neubiorev.2007.12.001 18295885

[34] Franke AG Bagusat C McFarlane C Tassone-Steiger T Kneist W Lieb K. The use of caffeinated substances by surgeons for cognitive enhancement. Ann Surg. (2014) 2014:830. 10.1097/SLA.0000000000000830 25072440

[35] Franke AG Christmann M Bonertz C Fellgiebel A Huss M Lieb K. Use of coffee, caffeinated drinks and caffeine tablets for cognitive enhancement in pupils and students in Germany. Pharmacopsychiatry. (2011) 44:331–8. 10.1055/s-0031-1286347 21993866

[36] Sharif S Guirguis A Fergus S Schifano F. The use and impact of cognitive enhancers among university students: a systematic review. Brain Sci. (2021) 11:30355. 10.3390/brainsci11030355 33802176 PMC8000838

[37] Brumboiu I Porrovecchio A Peze T Hurdiel R Cazacu I Mogosan C . Neuroenhancement in French and Romanian University Students, motivations and associated factors. Int J Environ Res Public Health. (2021) 18:83880. 10.3390/ijerph18083880 33917251 PMC8068007

[38] Pighi M Pontoni G Sinisi A Ferrari S Mattei G Pingani L . Use and propensity to use substances as cognitive enhancers in Italian Medical Students. Brain Sci. (2018) 8:110197. 10.3390/brainsci8110197 30423911 PMC6266090

[39] Nestle M. Soft drink “pouring rights”: marketing empty calories to children. Public Health Rep. (2000) 115:308–19. 10.1093/phr/115.4.308 11059423 PMC1308570

[40] Preedy V. Caffeine - Chemistry, Analysis, Function and Effects. London: Royal Society of Chemistry (2015)

[41] Lorenzo Calvo J Fei X Dominguez R Pareja-Galeano H. Caffeine and cognitive functions in sports: a systematic review and meta-analysis. Nutrients. (2021) 13:30868. 10.3390/nu13030868 33800853 PMC8000732

[42] Cappelletti S Piacentino D Sani G Aromatario M. Caffeine: cognitive and physical performance enhancer or psychoactive drug? Curr Neuropharmacol. (2015) 13:71–88. 10.2174/1570159X13666141210215655 26074744 PMC4462044

[43] Aggarwal R Mishra A Crochet P Sirimanna P Darzi A. Effect of caffeine and taurine on simulated laparoscopy performed following sleep deprivation. Br J Surg. (2011) 98:1666–72. 10.1002/bjs.7600 21761394

[44] Killgore WD Rupp TL Grugle NL Reichardt RM Lipizzi EL Balkin TJ. Effects of dextroamphetamine, caffeine and modafinil on psychomotor vigilance test performance after 44 H of continuous wakefulness. J Sleep Res. (2008) 17:309–21. 10.1111/j.1365-2869.2008.00654.x 18522689

[45] Wesensten NJ Killgore WD Balkin TJ. Performance and alertness effects of caffeine, dextroamphetamine, and modafinil during sleep deprivation. J Sleep Res. (2005) 14:255–66. 10.1111/j.1365-2869.2005.00468.x 16120100

[46] Repantis D Bovy L Ohla K Kuhn S Dresler M. Cognitive enhancement effects of stimulants: a randomized controlled trial testing methylphenidate, modafinil, and caffeine. Psychopharmacology. (2021) 238:441–51. 10.1007/s00213-020-05691-w 33201262 PMC7826302

[47] Franke AG Gransmark P Agricola A Schuhle K Rommel T Sebastian A . Methylphenidate, modafinil, and caffeine for cognitive enhancement in chess: a double-blind, randomised controlled trial. Eur Neuropsychopharmacol. (2017) 27:248–60. 10.1016/j.euroneuro.2017.01.006 28119083

[48] Ehlert AM Wilson PB. Stimulant use as a fatigue countermeasure in aviation. Aerosp Med Hum Perform. (2021) 92:190–200. 10.3357/AMHP.5716.2021 33754977

[49] Repantis D Schlattmann P Laisney O Heuser I. Modafinil and methylphenidate for neuroenhancement in healthy individuals: a systematic review. Pharmacol Res. (2010) 62:187–206. 10.1016/j.phrs.2010.04.002 20416377

[50] Bagot KS Kaminer Y. Efficacy of stimulants for cognitive enhancement in non-attention deficit hyperactivity disorder youth: a systematic review. Addiction. (2014) 109:547–57. 10.1111/add.12460 24749160 PMC4471173

[51] Bukstein O. Substance abuse in patients with attention-deficit/hyperactivity disorder. Medscape J Med. (2008) 10:24. 18324334 PMC2258479

[52] Kollins SH. A qualitative review of issues arising in the use of psycho-stimulant medications in patients with adhd and co-morbid substance use disorders. Curr Med Res Opin. (2008) 24:1345–57. 10.1185/030079908X280707 18384709

[53] Kollins SH MacDonald EK Rush CR. Assessing the abuse potential of methylphenidate in nonhuman and human subjects: a review. Pharmacol Biochem Behav. (2001) 68:611–27. 10.1016/S0091-3057(01)00464-6 11325419

[54] Van Puyvelde M Van Cutsem J Lacroix E Pattyn N. A state-of-the-art review on the use of modafinil as a performance-enhancing drug in the context of military operationality. Mil Med. (2022) 187:52–64. 10.1093/milmed/usab398 34632515

[55] Kelley AM Webb CM Athy JR Ley S Gaydos S. Cognition enhancement by modafinil: a meta-analysis. Aviat Space Environ Med. (2012) 83:685–90. 10.3357/ASEM.3212.2012 22779312

[56] Hart CL Haney M Vosburg SK Comer SD Gunderson E Foltin RW. Modafinil attenuates disruptions in cognitive performance during simulated night-shift work. Neuropsychopharmacology. (2006) 31:1526–36. 10.1038/sj.npp.1300991 16395298

[57] Sugden C Housden CR Aggarwal R Sahakian BJ Darzi A. Effect of pharmacological enhancement on the cognitive and clinical psychomotor performance of sleep-deprived doctors: a randomized controlled trial. Ann Surg. (2012) 255:222–7. 10.1097/SLA.0b013e3182306c99 21997802

[58] Killgore WD Kahn-Greene ET Grugle NL Killgore DB Balkin TJ. Sustaining executive functions during sleep deprivation: a comparison of caffeine, dextroamphetamine, and modafinil. Sleep. (2009) 32:205–16. 10.1093/sleep/32.2.205 19238808 PMC2635585

[59] Mutschler E Geisslinger G Kroemer HK Menzel S Ruth P. Arzneimittelwirkungen. Pharmakologie, Klinische Pharmakologie, Toxikologie. 10. Aufl. Stuttgart: Wissenschaftliche Verlagsgesellschaft (2012). p. 179.

[60] Franke AG Heinrich I Lieb K Fellgiebel A. The use of Ginkgo biloba in healthy elderly. Age. (2014) 36:435–44. 10.1007/s11357-013-9550-y 23736956 PMC3889903

[61] Diamond BJ Bailey MR. Ginkgo biloba: indications, mechanisms, and safety. Psychiatr Clin North Am. (2013) 36:73–83. 10.1016/j.psc.2012.12.006 23538078

[62] Birks J Grimley Evans J. Ginkgo biloba for cognitive impairment and dementia. Cochrane Database Syst Rev. (2007) 2:CD003120. 10.1002/14651858.CD003120.pub2 17443523

[63] Birks J Grimley Evans J. Ginkgo biloba for cognitive impairment and dementia. Cochrane Database Syst Rev. (2009) 1:CD003120. 10.1002/14651858.CD003120.pub3 19160216 PMC13076002

[64] Tan MS Yu JT Tan CC Wang HF Meng XF Wang C . Efficacy and adverse effects of ginkgo biloba for cognitive impairment and dementia: a systematic review and meta-analysis. J Alzheimers Dis. (2015) 43:589–603. 10.3233/JAD-140837 25114079

[65] Lewis JE Poles J Shaw DP Karhu E Khan SA Lyons AE . The effects of twenty-one nutrients and phytonutrients on cognitive function: a narrative review. J Clin Transl Res. (2021) 7:575–620. 34541370 PMC8445631

[66] Opitz-Welke A Lehmann M Seidel P Konrad N. Medicine in the penal system. Dtsch Arztebl Int. (2018) 115:808–14. 10.3238/arztebl.2018.0808 30642429 PMC6365676

[67] von Bernuth K Seidel P Krebs J Lehmann M Neumann B Konrad N . Prevalence of opioid dependence and opioid agonist treatment in the Berlin custodial setting: a cross-sectional study. Front Psychiatry. (2020) 11:794. 10.3389/fpsyt.2020.00794 32903474 PMC7435061

[68] Moore KE Roberts W Reid HH Smith KMZ Oberleitner LMS McKee SA. Effectiveness of medication assisted treatment for opioid use in prison and jail settings: a meta-analysis and systematic review. J Subst Abuse Treat. (2019) 99:32–43. 10.1016/j.jsat.2018.12.003 30797392 PMC6391743

[69] Wienke A. Der Arzt am Beginn des 21. Jahrhunderts - Zwischen Hippokrates und Staatsmedizin. In:Dierks C Wienke A, editors, *Zwischen Hippokrates und Staatsmedizin. Der Arzt am Beginn des 21. Jahrhunderts*. Berlin; Heidelberg: Springer-Verlag (2008). p. 149. 10.1007/978-3-540-77849-3_14 18064429

[70] Eberbach WH. Möglichkeiten und rechtliche Beurteilung der Verbesserung des Menschen - Ein Überblick. In:Wienke A Eberbach WH Kraemer HJ Janke K, editors, *Die Verbesserung des Menschen - Tatsächliche und rechtliche Aspekte der wunscherfüllenden Medizin*. Berlin; Heidelberg: Springer-Verlag (2009). p. 13. 10.1007/978-3-642-00883-2_1

[71] Schulz S Steigleder K Fangerau H Paul NW. Geschichte, Theorie und Ethik der Medizin. Berlin: Suhrkamp-Verlag (2006). p. 15.

[72] Stuttgart Higher Regional Court. Judgment 12/18/2012, 1 Ss 559/12, Neue Zeitschrift für Wirtschafts-, Steuer- und Unternehmensstrafrecht (NZWiSt). Stuttgart: Stuttgart Higher Regional Court (2013). p. 352– 4.

[73] German Federal Court of Justice. Decision of 10/26/2005, GSSt 1/05, Decisions of the Federal Court of Justice in Criminal Matters (BGHSt) 50. Karlsruhe: German Federal Court of Justice (2005). p. 252.

[74] Lindner JF. Fremdbestimmung durch Selbstbestimmung – Die82 Entscheidungsalternative‘ als Grundrechtsproblem. Archiv des öffentlichen Rechts (AöR). Tübingen: Mohr Siebeck GmbH & Co. KG (2015). p. 543–4. 10.1628/000389116X14525976022289

[75] Bavarian Supreme Court. Judgment of 10/28/1997, 4 St RR 221-974, Neue Juristische Wochenschrift (NJW). München: Bavarian Supreme Court (1998). p. 3580.

[76] Duttge G. § 263, Rn. 6. In:Dölling D Duttge G König S, editors, *Gesamtes Strafrecht § 263, Rn 6 4 Aufl*. Baden-Baden: Nomos-Verlag (2017).

[77] Hefendehl R. § 263, Rn. 88 ff. In:Joecks W Miebach K, editors, *Münchener Kommentar zum Strafgesetzbuch Band 5 2 Aufl*. München: CH Beck (2014).

[78] Tiedemann K. § 263, Rn. 93. In:Laufhütte HW Rissing-van Saan R Tiedemann K, editors, *Strafgesetzbuch – Leipziger Kommentar. 9. Band, 1. Teilband. 12. Aufl*. Berlin: Solon Buchservice.

[79] Dannecker G. § 263, Rn. 59. In:Graf JP Jäger M Wittig P, editors, *Wirtschafts- und Steuerstrafrecht 2 Aufl*. München: CH Beck (2017).

[80] German Federal Court of Justice. Judgment of 4/24/1952, 4 StR 854/51, Decisions of the Federal Court of Justice in Criminal Matters (BGHSt) 2. Karlsruhe: German Federal Court of Justice (1952). p. 325–6.

[81] German Federal Court of Justice. Decision of 02/18/1999, 5 StR 193-98, Decisions of the Federal Court of Justice in Criminal Matters (BGHSt) 45. Karlsruhe: German Federal Court of Justice (1999). p. 1–4.

[82] German Federal Constitutional Court. Decision of 01/17/1978, 1 BvL 13/76, Decisions of the Federal Constitutional Court (BVerfGE) 47. Karlsruhe: German Federal Constitutional Court (1978). p. 109.

[83] Freund G. §§ 1-4 Rn. 1 ff AntiDopG. In:Joecks W Miebach K, editors. Münchener Kommentar StGB 3 Aufl. München: CH Beck (2018).

[84] Kotz P Oglakcioglu MT. § 29 BtMG, Rn. 1217. In:Joecks W Miebach K, editors, *Münchener Kommentar zum Strafgesetzbuch. Band 6, Nebenstrafrecht I. 3. Aufl*. München: CH Beck (2017).

[85] German Federal Court of Justice. Judgment of 1/28/2014, 1 StR 494/13, Neue Juristische Wochenschrift (NJW). Karlsruhe: German Federal Court of Justice (2014). p. 1680.

[86] Altendorfer R. Medizinrecht – Betäubungsmittel-Verschreibung. Diabetes News. (2016) 15:7–11.

[87] Stuttgart Higher Regional Court. Judgment 12/18/2012, 1 Ss 559/12, Neue Zeitschrift für Wirtschafts-, Steuer- und Unternehmensstrafrecht (NZWiSt). Stuttgart: Stuttgart Higher Regional Court (2013). p. 352–4.

[88] Rehmann WA. AMG, § 95 AMG, Rn. 13. In:Rehmann WA Greve K, editors, *Arzneimittelgesetz 4 Aufl*. München: CH Beck (2014).

[89] Franke U Wienroeder K. § 29, Rn. 22. In:Franke U Wienroeder K, editors, *Betäubungsmittelgesetz 3 Aufl*. Berlin; Heidelberg: Springer-Verlag (2008).

[90] German Federal Court of Justice. Decision of 10/26/2005, GSSt 1/05, Decisions of the Federal Court of Justice in Criminal Matters (BGHSt) 50. Karlsruhe: German Federal Court of Justice (2005). p. 252.

[91] Lüneburg Higher Regional Court. Decision of 04/23/2012, 8 LA 45/11, Beck-Rechtsprechung (BeckRS). Lüneburg: Lüneburg Higher Regional Court (2012). p. 49890.

[92] Beubler E. Psychotrope Substanzen. In:Beubler E Haltmayer H Springer A, editors, *Opiatabhängigkeit – Interdisziplinäre Aspekte für die Praxis*. Berlin; Heidelberg: Springer-Verlag (2003). p. 134. 10.1007/978-3-7091-3796-3

[93] Dominik P. Stafbarkeit von pharmakologischem Neuroenhancement zur kognitiven Leistungssteigerung. Bern: Peter Lang GmbH Internationaler Verlag der Wissenschaften (2019). 10.3726/b16209

[94] Brantigan CO Brantigan TA Joseph N. Effect of beta blockade and beta stimulation on stage fright. Am J Med. (1982) 72:88–94. 10.1016/0002-9343(82)90592-7 6120650

